# A Randomized Experiment of Mailed Incentives to Improve Emergency Physician Survey Participation

**DOI:** 10.1111/acem.70385

**Published:** 2026-07-30

**Authors:** Steven C. Marcus, Mark Olfson, Sara Wiesel Cullen, Nicholas Cardamone, Rebecca Del Rossi, Raina M. Merchant, Tony Liu, Timothy Schmutte, Ming Xie, Nathaniel J. Williams

**Affiliations:** ^1^ School of Social Policy & Practice University of Pennsylvania Philadelphia Pennsylvania USA; ^2^ Department of Psychiatry, Vagelos College of Physicians and Surgeons Columbia University and the New York State Psychiatric Institute New York City New York USA; ^3^ Division of General Internal Medicine and Health Services Research David Geffen School of Medicine at UCLA Los Angeles California USA; ^4^ Department of Emergency Medicine Perelman School of Medicine, University of Pennsylvania Philadelphia Pennsylvania USA; ^5^ Department of Computer Science Mount Holyoke College South Hadley Massachusetts USA; ^6^ Department of Psychiatry Yale University School of Medicine New Haven Connecticut USA; ^7^ Department of Psychiatry, Perelman School of Medicine University of Pennsylvania Philadelphia Pennsylvania USA; ^8^ School of Social Work Boise State University Boise Idaho USA

**Keywords:** emergency physicians, monetary incentives, physician surveys, research recruitment, response rates

Emergency physicians are frequently asked to provide feedback on clinical practice, quality improvement, and implementation efforts. Physician surveys remain one of the most efficient methods for obtaining such feedback, yet response rates among physicians continue to decline [1]. Emergency physicians may be particularly challenging to engage because of shift‐based schedules, high clinical workloads, and administrative overload [2], all of which limit time available for research participation. Although several studies have evaluated strategies to improve physician survey participation [3, 4, 5, 6], limited randomized evidence exists regarding effective approaches for recruiting emergency physicians who have failed to respond to repeated emailed survey invitations. Social exchange theory suggests that unconditional prepaid incentives may increase survey participation by invoking the norm of reciprocity, whereby recipients feel an obligation to return a favor [7]. Providing a tangible representation of a promised incentive, such as the unloaded payment card that will later be used for compensation, may also increase participation by enhancing the salience and credibility of the future reward. The objective of this study was to evaluate these two mailed incentive strategies, alone and in combination, for increasing participation among emergency physician survey nonresponders.

As part of a larger NIH‐funded study evaluating an electronic health record‐integrated dashboard designed to support suicide risk assessment in emergency departments, we conducted a national survey of emergency physicians. We invited a random sample of 280 emergency physicians selected from the American Medical Association (AMA) Physician Masterfile to complete an 11‐minute web‐based questionnaire. The sample was obtained through Redi‐Data, an AMA‐licensed provider of physician data, which supplied Masterfile mailing addresses and appended email addresses. Physicians received an initial email invitation followed by five reminder emails over approximately 5 months. The wording of the invitation emails varied across contacts, but each emphasized the study's NIH funding, national importance, and the $200 completion incentive. Despite these efforts, only 20 of 280 physicians (7.1%) completed the survey.

To improve participation among the nonresponders, we developed personalized mailed recruitment materials. The mailing followed Dillman's tailored design recommendations [8] and featured a hand‐signed, individually addressed, professionally designed, one‐page letter that described the study purpose, NIH funding, university sponsorship, survey length, $200 survey compensation, and options to participate via either a QR code or direct web link (see Methods Supplement accompanying the online article). Mailings were sent in a first‐class envelope bearing a commemorative stamp and the investigator's handwritten surname above the university return address to increase salience and legitimacy.

After excluding physicians without verified home mailing addresses (*n* = 60), the remaining nonresponders (*n* = 200) were randomized in a 2 × 2 factorial design evaluating two recruitment strategies: inclusion of an unloaded VISA debit card that would be loaded upon survey completion and inclusion of an unconditional $2 bill as a token of appreciation. The invitation letter in all groups described the $200 survey incentive. Physicians were assigned to receive: (1) the invitation letter alone, (2) the letter plus the unloaded VISA debit card, (3) the letter plus the $2 bill, or (4) the letter plus both incentive components. Mailers were sent in March 2026 after home postal addresses were verified. The primary outcome was survey completion during the subsequent 4‐week study period.

Pairwise comparisons of response rates between study groups were conducted using 2 × 2 contingency table analyses with the *csi* command in Stata [9] to estimate risk ratios (RRs) and corresponding *p*‐values. All tests were two‐sided, and *p* < 0.05 were considered statistically significant. The [blinded] Institutional Review Board determined the study to be exempt from review.

Among the 200 physicians who had not responded to the initial email recruitment campaign, 47 completed the survey (23.5%). Response rates were 8.8% with the invitation alone, 23.3% with the invitation plus an unloaded debit card, 35.6% with the invitation plus a $2 bill, and 29.1% with both items (Figure 1). Relative to the invitation alone, response rates were 2.65 times higher in the debit card group (*p* = 0.045), 4.05 times higher in the $2 bill group (*p* = 0.001), and 3.32 times higher in the combined incentive group (*p* = 0.006). No significant differences were observed among the three incentive conditions (*p* values 0.206–0.516).

**FIGURE 1:**
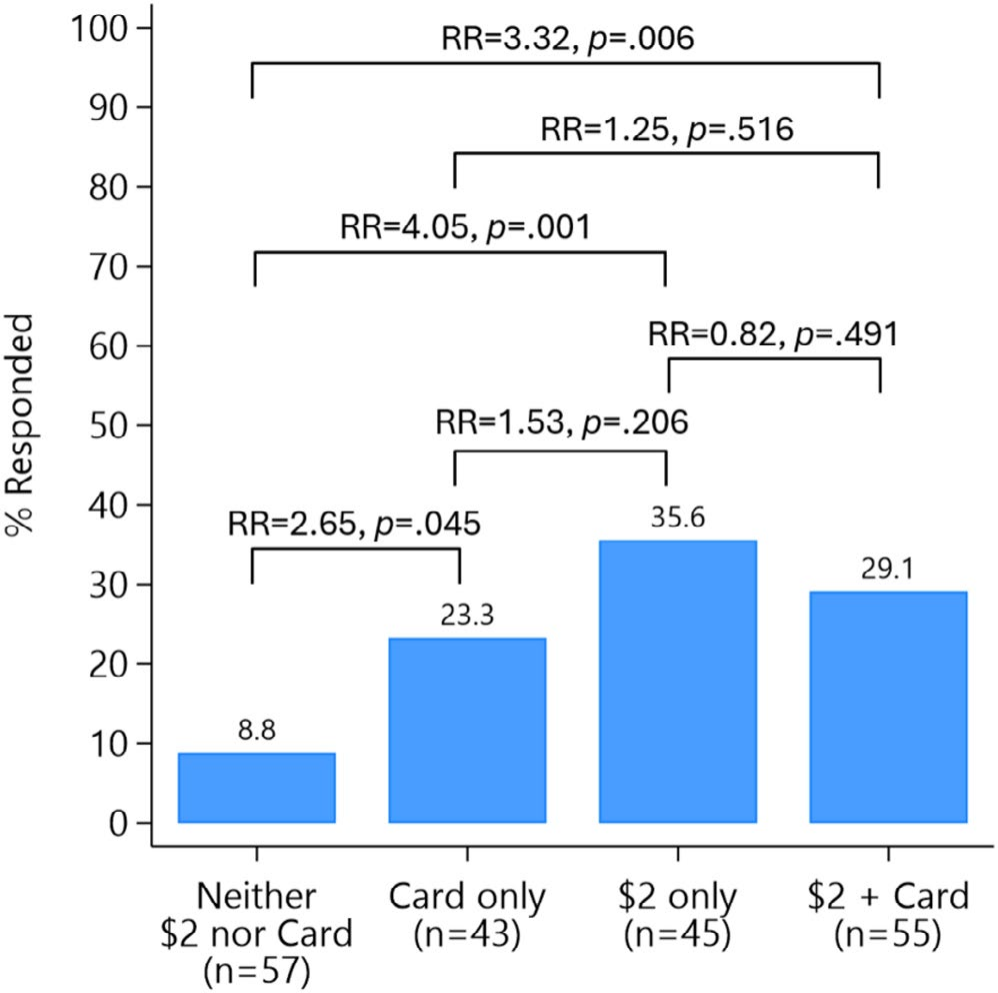
Response rates among emergency physician survey nonresponders by randomized incentive condition.

A nominal unconditional $2 incentive outperformed a strategy designed to increase the salience of a much larger promised $200 payment. This finding is consistent with social exchange theory and the norm of reciprocity [10], suggesting that the timing and unconditional nature of an incentive may be more influential than its monetary value among physicians who have already declined repeated electronic invitations. Previous research, including a systematic review of clinician surveys, has shown that unconditional monetary incentives increase physician survey response rates [11]. Our findings extend this literature by suggesting that even a nominal prepaid incentive may be more effective than reinforcing an already substantial promised reward.

Several limitations should be considered. The study involved a relatively small sample of physicians and focused on a survey related to suicide risk assessment, which may limit generalizability to other survey topics. All physicians were offered a $200 completion incentive, and therefore the observed effects reflect the incremental impact of the mailed incentives. The novelty of receiving a $2 bill through the mail may also have contributed to its effectiveness. Results may differ in surveys offering smaller or different rewards. The findings apply specifically to physicians who did not respond to repeated email recruitment and may not generalize to physicians approached during initial survey recruitment. Finally, because all participants received the same mailed recruitment package, we were unable to isolate the effects of individual design elements, such as the one‐page format, handwritten components, or QR‐code access.

The findings provide experimental evidence that when emergency medicine physicians do not respond to repeated electronic survey requests, a small unconditional cash token produced higher response rates than increasing the salience of a large, promised reward. Among physicians who had ignored five email invitations despite a $200 completion incentive, inclusion of a $2 bill increased response rates more than fourfold. Hybrid recruitment strategies combining electronic invitations with small prepaid mailed incentives may improve response rates in physician surveys.

## Funding

This research was funded by the National Institute of Mental Health, National Institutes of Health, grant R01MH126895. The content is solely the responsibility of the authors and does not necessarily represent the official views of the National Institutes of Health.

## Disclosure

Role of the Sponsor: The sponsors had no role in the design, analysis, interpretation, or publication of this study.

## Conflicts of Interest

The authors declare no conflicts of interest.

## Supporting information


**Data S1:** Methods supplement recruitment mailing.

## Data Availability

The data that support the findings of this study are available from the corresponding author upon reasonable request.
