# Supplementary material for: A Randomized Experiment of Mailed Incentives to Improve Emergency Physician Survey Participation
Source: Acad Emerg Med. 2026 Jul 30;33(7):e70385. doi: 10.1111/acem.70385 (PMC13424852; doi:10.1111/acem.70385)
Supplement: Supplementary file 1 — Data S1: Methods supplement recruitment mailing. [file ACEM-33-0-s001.pdf]

Methods Supplement (Recruitment Mailing)

Penn
University of Pennsylvania

Help Us Improve
Suicide Risk Assessment in Emergency Care

11-Minute Survey | $200 Honorarium

Dear Dr. X,

You are invited to participate in an NIH-funded study evaluating an EHR dashboard designed to help emergency physicians identify patients at risk for suicide.

We are asking for 11-minutes of your time to evaluate the dashboard through a brief, web-based survey. Your feedback will help strengthen this suicide prevention tool designed for physicians, by physicians.

AS A THANK YOU:

$2 bill enclosed
as a token of appreciation

$200 Honorarium
upon survey completion*

–OR–

$200 donation
in your name to the American Foundation for Suicide Prevention

WHY YOUR PARTICIPATION MATTERS

- Improve suicide prevention
- Inform real-world EHR tools
- Support your colleagues & patients

COMPLETE THE SURVEY

01 **Visit this site:** https://tinyurl.com/UPennED2026

02 **Enter your ID:** XXXX

03 **Complete survey**

–OR–

Scan QR and complete survey

Thank you in advance for your contribution to suicide prevention efforts in emergency care. We hope you will complete the survey today.

Raina M. Merchant, MD, MSHP
University of Pennsylvania
Department of Emergency Medicine

Mark Olfson, MD, MPH
Columbia University
Department of Psychiatry

Steven C. Marcus, Ph.D.
University of Pennsylvania
School of Social Policy and Practice

www.sp2.upenn.edu questions? edstudy@pennmedicine.upenn.edu

NIH Funded Study

** Visa debit card enclosed will be activated upon completion.*
